# Outbreak of viral hemorrhagic fever caused by dengue virus type 3 in Al-Mukalla, Yemen

**DOI:** 10.1186/1471-2334-13-136

**Published:** 2013-03-14

**Authors:** Tariq A Madani, El-Tayeb ME Abuelzein, Hussein MS Al-Bar, Esam I Azhar, Moujahed Kao, Haj O Alshoeb, Alabd R Bamoosa

**Affiliations:** 1 Department of Medicine, Faculty of Medicine, King Abdulaziz University, PO Box 80215, Jeddah, 21589, Saudi Arabia; 2 Scientific Chair of Sheikh Mohammad Hussein Alamoudi for Viral Hemorrhagic Fever, King Abdulaziz University, Jeddah, Saudi Arabia; 3 Special Infectious Agents Unit, King Fahd Medical Research Center, King Abdulaziz University, Jeddah, Saudi Arabia; 4 Department of Family and Community Medicine, Faculty of Medicine, King Abdulaziz University, Jeddah, Saudi Arabia; 5 Department of Medical Laboratory Technology, Faculty of Applied Medical Sciences, King Abdulaziz University, Jeddah, Saudi Arabia; 6 Ministry of Health, Al-Mukalla, Yemen

**Keywords:** Dengue virus type 3, Viral hemorrhagic fever, Outbreak, Al-Mukalla, Yemen

## Abstract

**Background:**

Investigations were conducted by the authors to explore an outbreak of viral hemorrhagic fever (VHF) reported in 2010 from Al-Mukalla city, the capital of Hadramout in Yemen.

**Methods:**

From 15–17 June 2010, the outbreak investigation period, specimens were obtained within 7 days after onset of illness of 18 acutely ill patients hospitalized with VHF and 15 household asymptomatic contacts of 6 acute cases. Additionally, 189 stored sera taken from acutely ill patients with suspected VHF hospitalized in the preceding 12 months were obtained from the Ministry of Health of Yemen. Thus, a total of 222 human specimens were collected; 207 specimens from acute cases and 15 specimens from contacts. All samples were tested with RT-PCR for dengue (DENV), Alkhumra (ALKV), Rift Valley Fever (RVFV), Yellow Fever (YFV), and Chikungunya (CHIKV) viruses. Samples were also tested for DENV IgM, IgG, and NS1-antigen. Medical records of patients were reviewed and demographic, clinical, and laboratory data was collected.

**Results:**

Of 207 patients tested, 181 (87.4%) patients were confirmed to have acute dengue with positive dengue NS1-antigen (97 patients, 46.9%) and/or IgM (163 patients, 78.7%). Of the 181 patients with confirmed dengue, 100 (55.2%) patients were IgG-positive. DENV RNA was detected in 2 (1%) patients with acute symptoms; both samples were molecularly typed as DENV type 3. No other VHF viruses were detected. For the 15 contacts tested, RT-PCR tests for the five viruses were negative, one contact was dengue IgM positive, and another one was dengue IgG positive. Of the 181 confirmed dengue patients, 120 (66.3%) patients were males and the median age was 24 years. The most common manifestations included fever (100%), headache (94.5%), backache (93.4%), malaise (88.4%), arthralgia (85.1%), myalgia (82.3%), bone pain (77.9%), and leukopenia (76.2%). Two (1.1%) patients died.

**Conclusions:**

DENV-3 was confirmed to be the cause of an outbreak of VHF in Al-Mukalla. It is important to use both IgM and NS1-antigen tests to confirm acute dengue particularly under the adverse field conditions, where proper storage and transportation of specimens are missing, which substantially reduce the sensitivity of the RT-PCR for detecting DENV RNA.

## Background

The viruses that cause viral hemorrhagic fever (VHF) belong to four different families: *Bunyaviridae*, *Flaviviridae, Filoviridae,* and *Arenaviridae.* Many countries of the Middle East have experienced resurgent outbreaks of several VHFs including dengue virus (DENV), Rift Valley Fever (RVF), Crimean-Congo Hemorrhagic Fever (CCHF), and Alkhumra virus (ALKV) [1-7]. Additionally, Chikungunya (CHIKV), a non-hemorrhagic acute mosquito-borne viral illness that often mimics dengue fever, has caused outbreaks in many Asian countries beyond its original boundaries in Africa [8]. Some of these VHFs are endemic in the region [2]. Even though several studies have documented outbreaks and endemic transmission of dengue, ALKV, RVF, and CCHF in Saudi Arabia, very scarce data have been published on VHF in the neighboring country, Yemen [9-12].

In the period, 15–17 June 2010, investigations were conducted by the authors to explore an outbreak of viral hemorrhagic fever that was reported from Al-Mukalla city, the capital of Hadramout in Yemen. This study summarizes the results of this outbreak investigation.

## Methods

### The outbreak region: Al-Mukalla, Hadrahmout, Yemen

Hadramout is the largest governorate in the republic of Yemen. It lies in the south of Yemen along the Gulf of Aden in the Arabian Sea (15.9°N; 49°E) and extends eastwards to the borders of Dhofar region of Oman. It has a diverse topography with coastal plains along the Arabian sea, mountains and hills reaching 2000 meters above sea level, and the extensive desert of the empty quarter, the largest sand desert in the world. Many valleys, known as wadis, run through Hadramout. The biggest of which is Wadi Hadramout which has several branches. The climate in Hadramout is tropical hot in the summer with temperatures up to 40°C. However, the coastal area is moderate in temperature due to blow of the humid monsoon trader winds with temperatures up to 36°C. In the winter, the temperature drops to 20-24°C in the coast and to 17-20°C in the interior parts. Al-Mukalla, is the main Sea Port and the capital city of Hadramout (14°32’N; 49°08’E). It is 480 km east of Aden and 777 km from the capital Sanaa. It is the fourth largest city in Yemen, with an area of 193,032 km^2^. The total population is approximately 300,000 people. Health care facilities in the coastal plain of Hadramout include 13 hospitals and 25 primary health care centers.

### Case definition

The case definition developed by Madani was adapted to identify patients with VHF [5]. Viral hemorrhagic fever was clinically suspected if a patient had an acute febrile illness of at least 2 days duration and at least 2 of the following 5 clinical or laboratory criteria: (1) headache, retro-orbital pain, arthralgia, myalgia, bone pain, backache, or rash; (2) at least 3-fold elevation of alanine transferase (AlT), or aspartate transferase (AsT), or clinical jaundice; (3) features of encephalitis such as confusion, disorientation, drowsiness, coma, neck stiffness, hemiparesis, paraparesis, or convulsions; (4) hemorrhagic manifestations such as ecchymosis, purpura, petechiae, gastrointestinal bleeding (hematemesis, melena, hematochezia), epistaxis, bleeding from puncture sites, or menorrhagia; (5) leukopenia (white blood cells count < 4.5 × 10^9^/L), thrombocytopenia (platelet count < 100 x10^9^/L), or lactate dehydrogenase (LDH) or creatine phosphokinase (CK) enzyme > 2 times upper normal level (>500 and >400 U/L, respectively).

### Specimens obtained

As part of standard patient care, blood specimens were collected within 7 days after onset of illness from all patients with clinically suspected VHF who were admitted to hospitals in Al-Mukalla from 15–17 June 2010. Blood was also collected from some of the household contacts of the hospitalized patients after obtaining verbal informed consents. Stored serum samples which were collected in the preceding 12 months from acutely febrile patients with suspected VHF were obtained from the Ministry of Health of Yemen. The specimens were transported in IATA compliant transport containers on dry ice to the special Infectious Agents Units, a biosafety level-3 virology laboratory, at King Fahad Medical Research Centre, King Abdulaziz University, Jeddah, Saudi Arabia.

### Data collection

Medical records of patients hospitalized with clinically suspected VHF were reviewed and data recorded on a standard case report form. Information collected included patient demographics, clinical manifestations, laboratory results, and outcome.

### Laboratory confirmation

A simplified single-tube multiplex reverse transcriptase-polymerase chain reaction (RT-PCR) was used to detect dengue virus and determine its type as described by Lanciotti et al. [13]. Briefly, 5 μl of extracted RNA were added to 20 μl of an RT-PCR mixture consisting of 5 μl of 5x QIAGEN One Step RT-PCR Buffer, 1 μl of dNTPs, 1.0 μl of RT-PCR Enzyme Mix, 1 mM of the 5’ primer D1 and 3’ primers D2 and TS1, 0.5 mM of each of the 3´ primers TS2, TS3, and TS4. Reverse transcription was performed at 50°C for 30 minutes, followed directly by 40 cycles of amplification consisting of 94°C for 1 minute, 55°C for 1 minute, and 72°C for 1 minute, ending with a final extension of 72°C for 10 minutes. Ten μl of the product were analyzed by electrophoresis in a 1.5% agarose gel in 1X Tris Borate EDTA (TBE) buffer containing 4 ng/ml of ethidium bromide. The expected size of the amplified products was as follows: 482 bp for dengue-1, 119 bp for dengue-2, 290 bp for dengue-3, and 392 bp for dengue-4. Positive controls for the RT-PCR included RNA extracted from tissue culture isolates of different serotypes, which produces an amplicon of 511 bp with primers D1 and D2 and serves as an additional control for the molecular typing of dengue amplification process. The human serum samples were also tested with RT-PCR for ALKV, RVFV, YFV, and CHIKV using previously described methods [7,14,15].

### Detection of dengue IgM and IgG antibodies in the tested sera

The human serum samples were tested for the presence of dengue IgM and IgG antibodies employing capture ELISA Kits (Dengue Duo IgM & IgG, Panbio, Australia) as described by the manufacturer (http://panbiodengue.com). The Panbio dengue early capture ELISA employing the NS1- antigen (Inverness Medical Innovation Australia Pty Ltd, Australia), was employed to detect the NS1 dengue virus antigen in the tested serum samples according to the procedure described by the manufacturer.

### Veterinary assessment of livestock

Many families in Hadramout keep sheep and goats in the back-yards of their houses. There are also several animal market places in the capital city, Al-Mukalla. Blood specimens were randomly collected from animals in the back-yards of the suspected human cases and from other houses and market places. The animal species were sheep and goats of different age groups. All the animals were clinically examined by one of the authors (EMEA, a professor of Veterinary Medicine and Arbovirology) and the relevant epidemiological data was collected from the animals’ owners. Sheep and goats sera were tested with real time RT-PCR for detection of RVFV, ALKV and CHIKV RNA as described above. King Abdulaziz University's policy on the care and handling of animals was followed.

## Results

From 15–17 June 2010, the outbreak investigation period, specimens were obtained within 7 days after onset of illness of 18 acutely ill hospitalized patients who fulfilled the case definition for VHF and 15 household asymptomatic contacts of 6 acute cases. Additionally, 189 stored sera taken from acutely ill patients with suspected VHF hospitalized in the preceding 12 months were obtained from the Ministry of Health of Yemen. Thus, a total of 222 human specimens were collected, of which 207 specimens were from acute cases and 15 specimens were from contacts. DENV RNA was detected in two samples from 2 patients with acute symptoms; one of them was a stored specimen. Both of these two DENV-positive samples were molecularly typed as DENV type 3. One of the two RNA-positive patients was also NS1-antigen and IgM-positive, whereas the other one was negative for both of them. RT-PCR of the 222 human specimens was negative for ALKV, RVFV, CHIKV, and YFV RNA.

Table 1 shows the results of the RT-PCR for detection of DENV RNA and ELISA for detection of DENV NS1-antigen, IgM and IgG. Of 207 acute cases tested, 181 (87.4%) patients were confirmed to have acute dengue with positive dengue NS1-antigen and/or IgM. Ninety seven (46.9%) patients were positive for DENV NS1-antigen, and 163 (78.7%) patients were positive for DENV IgM. Of the 181 patients with confirmed dengue, 100 (55.2%) patients were IgG-positive. Table 2 summarizes the details of the diagnostic laboratory results among the 181 patients with confirmed dengue. Of the 97 patients with positive NS1-antigen, 79 (81.4%) patients were also positive for IgM. Of the 163 patients who were positive for IgM, 79 (48.5%) patients were also positive for NS1-antigen.

**Table 1:** RT-PCR for dengue virus RNA and ELISA results for detection of dengue virus NS1, IgM and IgG antibodies in human sera in Almukalla, Yemen

**Categories of tested groups**	**No. tested**	**No. of patients positive for dengue RNA (%)**	**No. of patients positive for NS1 antigen (%)**	**No. of patients positive for any dengue virus antibodies (%)**	**No. of patients positive for IgM, IgG, or both (%)**		
					**IgM alone (%)**	**IgG alone (%)**	**IgG & IgM (%)**
Acute cases	207	2 (1)	97 (46.9)*	169 (81.6)	69 (33.3)†	6 (2.9)‡	94 (45.4)§
Contacts	15	0	0	2 (13.3)	1 (6.7)	1 (6.7)	0

* Of these 97 NS1-positive patients, 79 (81.4%) patients were also positive for IgM.

† Of these 69 IgM-positive patients, 50 (72.5%) patients were also NS1-positive.

‡ Considered to have acute dengue and past exposure to another dengue serotype based on positive NS1 and IgG.

§ Of these 94 IgM/IgG-positive patients, 29 (30.9%) patients were also NS1-positive.

**Table 2:** Dengue NS1, IgM, and IgG results of 181 patients with confirmed dengue infection

**Patterns of laboratory results**	**Laboratory result**			**Number of patients (%)**
	**NS1**	**IgM**	**IgG**	
1	+	+	-	50 (27.6)
2	+	-	-	12 (6.6)
3	-	+	-	19 (10.5)
4	+	+	+	29 (16.0)
5	+	-	+	6 (3.3)
6	-	+	+	65 (35.9)
Total number (%)	97 (53.6)	163 (90.0)	100 (55.2)	181 (100)

Of the 181 confirmed cases, 120 (66.3%) patients were males. The mean age was 21 ± 12 (range: 3–75) years and the median was 24 years. Table 3 shows the clinical and laboratory manifestations of the 181 patients with confirmed acute dengue fever. Two (1.1%) patients died of complicated dengue infection.

**Table 3:** Clinical and laboratory features of 181 patients with laboratory-confirmed dengue fever in Al-Mukalla, Yemen, 2009-2010

**Clinical or laboratory feature**	**Number of patients (%)**
Fever	181 (100)
Headache	171 (94.5)
Backache	169 (93.4)
Malaise	160 (88.4)
Arthralgia	154 (85.1)
Myalgia	149 (82.3)
Bone pain	141 (77.9)
Leukopenia	138 (76.2)
Anorexia	112 (61.9)
Retro-orbital pain	108 (59.7)
Nausea	92 (50.8)
Thrombocytopenia	83 (45.9)
Vomiting	44 (24.3)
Abdominal pain	34 (18.9)
Hemorrhagic manifestations	14 (7.7)
Rash	9 (5.0)
Central nervous system manifestations	3 (1.7)
Altered sensorium	3 (1.7)
Neck stiffness	2 (1.1)
Convulsions	1 (0.6)
Jaundice	2 (1.1)

Forty seven goats and 6 sheep were clinically examined and found to be healthy. Sera from these animals were negative for the presence of RNA for ALKV, RVFV, and CHIKV.

## Discussion

The historic record of dengue infection in Yemen goes back to the 19^th^ century when a severe outbreak was reported in 1870 – 1873 [1]. In 1954 a dengue epidemic was also reported in Yemen, when 98% of the population in Alhudaydah was affected [1]. In 1983 a patient who had recently returned from South Yemen to the United Stated was confirmed to have dengue infection [16]. In 2003, a survey on 158 blood specimens from 158 febrile patients and 716 blood specimens from apparently healthy individuals, from various localities in Yemen, showed that all specimens were negative for dengue IgM and IgG [17]. In 2010, an Italian patient who had returned from Yemen to Italy was confirmed to have contracted dengue infection [18]. Interestingly, that patient acquired the infection from Al-Mukalla and the serotype was also DENV-3. Several other official reports described outbreaks of dengue in various localities of Yemen (unpublished data, Ministry of Health, Yemen). DENV-3 was confirmed to be the cause of the dengue outbreak described herein. Sequencing of the two positive samples, intended to be performed by the authors in the near future, will provide phylogenetic and epidemiological information about the virus.

The four distinct serotypes of DENV (DENV-1, DENV-2, DENV-3 and DENV-4) don’t cross-protect, but infection with one serotype gives life-long immunity against its homologous challenging field virus, i.e. the four serotypes are cross-reacting but not cross-neutralizing. In this study, more than half (55.2%) of the patients with acute dengue had serological evidence (positive for DENV IgG) of previous exposure to this virus suggesting that more than one serotype are circulating in the region, thus increasing the risk of complicated dengue through a phenomenon known as antibody-dependent enhancement or immune enhancement syndrome [19-21]. It should be noted that some of the IgG positive results of the 181 patients with acute dengue might have represented early development of IgG response at the end of the first week of acute dengue infection rather than previous exposure to another dengue serotype.

Rapid and early laboratory diagnosis of dengue infection is a pivotal step towards monitoring of the cases and earlier implementation of supportive therapy to prevent mortality from dengue haemorrhagic fever and dengue shock syndrome. The three rapid diagnostic tests currently available are: the RT-PCR, which can detect the dengue RNA within 1–5 days after onset of fever; the detection of the dengue virus non-structural protein 1 (NS1-antigen) within 1–9 days after onset of fever; and IgM detection at least 5–7 days after onset of fever. Therefore, RT-PCR and NS1-antigen tests can be diagnostic before IgM sero-conversion. The NS1-antigen is a 46–50 Kilo Dalton (K Da) glycoprotein found in dengue virus-infected cells in two forms: membrane associated (mNS1) and secreted (sNS1) form. Detection of the (sNS1) at high levels in plasma and sera of dengue patients was found to correlate well with viraemia level and the type of clinical dengue manifestations [22]. It was also found that (sNS1) was higher in patients who developed dengue hemorrhagic fever compared to those who developed classic dengue fever [22].

In this study, the use of NS1-antigen test identified 53.6% of the confirmed cases, whereas IgM test identified 90% of them. The use of NS1-antigen test identified 18.6% of patients who would have been missed if IgM test alone (without NS1-antigen test) were used to confirm acute dengue, whereas the use of IgM test identified 51.5% of patients who would have been missed if NS1-antigen test alone (without IgM test) were used to confirm acute dengue. The RT-PCR identified only 1.0% of the confirmed cases. This was most likely due to degradation of RNA during storage and shipping.

## Conclusions

DENV-3 was confirmed to be the cause of an outbreak of VHF in Al-Mukalla, Hadramout, Yemen in 2010. More than half of the affected patients had evidence of previous exposure to DENV indicating that more than one serogroup were circulating in the region. It is important to use both IgM and NS1-antigen tests to confirm acute dengue particularly under the adverse field conditions, where proper storage and transportation of specimens are missing, which substantially reduce the sensitivity of RT-PCR for detecting DENV RNA.

## Ethical approval

Ethical approval for the human and animal research and experiments was obtained from the Research Ethics Committee of the Faculty of Medicine, King Abdulaziz University, Jeddah, Saudi Arabia.

## Competing interests

The authors declare that they have no competing interests.

## Authors’ contributions

TAM was the principal investigator, conceived and designed the study and clinically assessed the patients; TAM, EMEA, HOA, and ARB collected the samples; EIA, and MK performed the real time RT-PCR; TAM, EMEA, HMSA, EIA, MK analyzed and interpreted the data; TAM, and EMEA wrote the manuscript; HMSA, EIA, MK, HOA, and ARB critically revised the manuscript. All authors read and approved the final manuscript.

## Pre-publication history

The pre-publication history for this paper can be accessed here:

http://www.biomedcentral.com/1471-2334/13/136/prepub
